# Osteoblastoma-like Osteosarcoma of the Cuboid: A Case Report

**DOI:** 10.1186/1749-799X-5-52

**Published:** 2010-08-06

**Authors:** Navin L Kumar, Andrew E Rosenberg, Kevin A Raskin

**Affiliations:** 1 Harvard Medical School, Boston, MA 02115, USA; 2 Harvard Medical School, Department of Pathology -- WRN 2, Massachusetts General Hospital, 55 Fruit Street, Boston, MA 02114, USA; 3 Harvard Medical School, Orthopaedic Oncology Service, Massachusetts General Hospital, 55 Fruit Street, Yawkey Outpatient Center, Suite 3B, Boston, MA 02114, USA

## Abstract

Osteosarcoma most commonly arises in the long bones of the skeleton, and rarely develops in the bones of the foot. We describe a patient who presented with left foot pain, whose radiographic evaluation revealed a lytic destructive mass in the cuboid bone. A biopsy showed an osteoblastoma-like variant of osteosarcoma and the patient was treated with preoperative chemotherapy and amputation. Osteosarcoma of the foot is uncommon and the literature reveals that it is often associated with a delay in diagnosis.

## Background

Osteosarcoma is a malignant neoplasm of bone in which tumor cells produce neoplastic bone matrix [1]. It is the most common sarcoma of bone, and follows multiple myeloma as the second most common primary malignancy of the skeleton [2]. Osteosarcoma usually affects patients in the late teenage years, and predominantly originates in the long bones, particularly around the knee [1]. Rarely, osteosarcoma develops in the bones of the foot [3]. This case report describes a patient who was found to have osteosarcoma of the cuboid bone after developing pain and swelling of his left foot. Although initial diagnostic tests suggested that the lesion was benign, an open biopsy revealed a high grade osteoblastoma-like variant of osteosarcoma, which to our knowledge is the first case published involving the cuboid bone.

## Case Presentation

### Case Report

**Figure 1:**
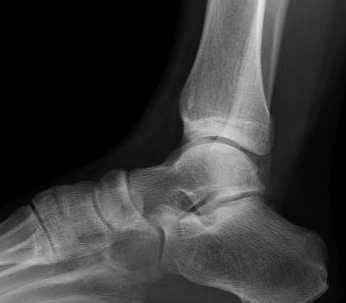
**Plain radiograph of the left foot**. The radiograph demonstrates a large lytic lesion of the left cuboid.

**Figure 2:**
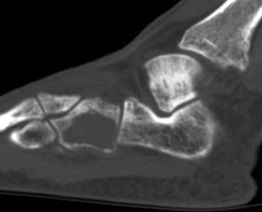
**Computed tomography (CT) of the left foot**. The CT scan demonstrates a 2-3 cm lytic lesion within the left cuboid, with extreme thinning of the overlying cortex and possible cortical interruption.

A 32 year old man presented to an outside hospital with a one month history of left foot pain. He recalled that the pain started after he had twisted his ankle during a round of golf. Since that time, the pain had persisted and was most severe along the lateral aspect of his left foot. On physical examination, there was moderate swelling over the lateral aspect of the hindfoot, with point tenderness directly over the cuboid bone. The remainder of the foot exam was normal. A plain radiograph of the left foot showed a large oval, cystic mass within the cuboid bone that had relatively well-defined margins but with obfuscation of the lateral cortex (figure 1). There was no significant periosteal reaction, and no soft tissue mass or calcifications were appreciated. A computed tomography (CT) scan showed an expansile lytic lesion, which was contained by an extremely thin shell of reactive bone (figure 2). The margins of the lesion were circumscribed, but not sclerotic, and there were no internal foci of mineralization. There was also no evidence of ankle effusion.

**Figure 3:**
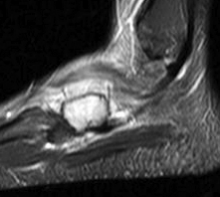
**Magnetic resonance imaging (MRI) of the left foot**. The MRI demonstrates the entire cuboid bone replaced with abnormal signal intensity. There is no associated soft tissue mass.

**Figure 4:**
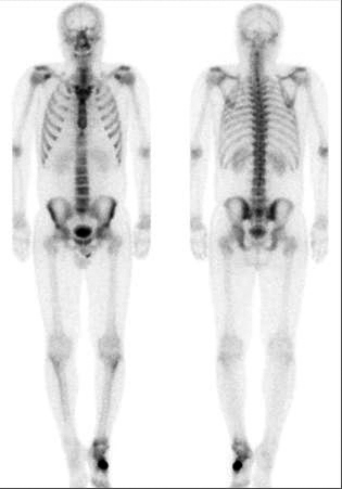
**A Tc99 M Bone Scan**. The bone scan demonstrates intense uptake localized to the left cuboid lesion, with no evidence of metastatic disease.

Magnetic resonance imaging (MRI) demonstrated a gadolinium-enhancing lesion, with increased signal intensity on the T2 weighted image (figure 3). The lesion demonstrated slight heterogeneous enhancement, and there was no associated pathologic fracture or soft tissue mass identified. There was, however, extensive edema in the adjacent soft tissues. A two-phase technetium-99m-methylene diphosphonate (Tc99 M MDP) was obtained and demonstrated marked uptake corresponding to the left cuboid lesion, with no other foci of significant uptake (figure 4). Based on the clinical and radiographic findings, the lesion was thought to represent a benign, but potentially locally aggressive neoplasm such as giant cell tumor of bone.

The patient's pain became progressively more severe and he no longer was able to weight bear. An open biopsy was performed and a frozen section revealed a giant cell rich lesion with atypical mononuclear stromal cells and areas of extracellular eosinophilic matrix. The differential diagnosis was an atypical giant cell tumor versus a variant of osteosarcoma. A discussion regarding immediate management concluded that it would be best to thoroughly curette the lesion and pack the defect with cement, which would stabilize the lateral column and be adequate therapy for a benign lesion. If, however, the histologic analysis of the additional tissue demonstrated an osteosarcoma, then an ampution would be performed at a later date and the curettage would not have compromised treatment for this type of tumor.

**Figure 5:**
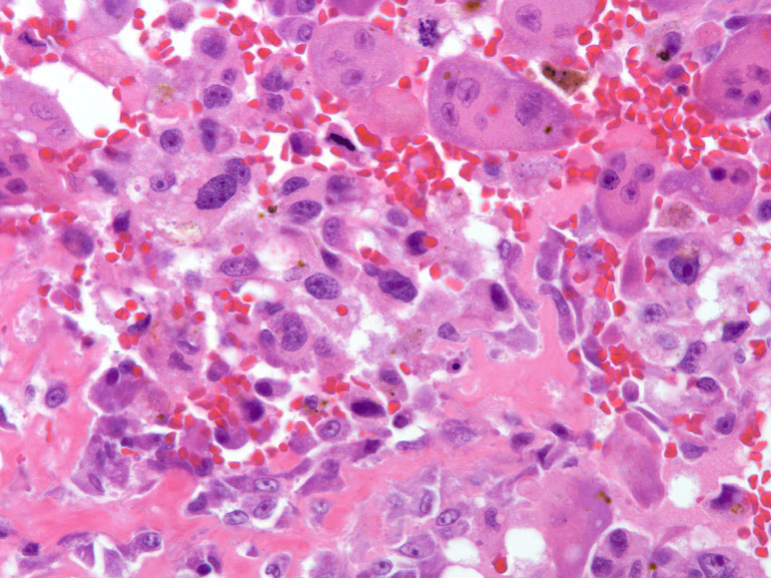
**Permanent section of the left cuboid resembling osteoblastoma**. Region of tumor resembling osteoblastoma composed of well formed interconnecting trabeculae of woven bone lined by prominen cytologically banal osteoblasts. The stroma consists of loose fibrovascular tissue.

**Figure 6:**
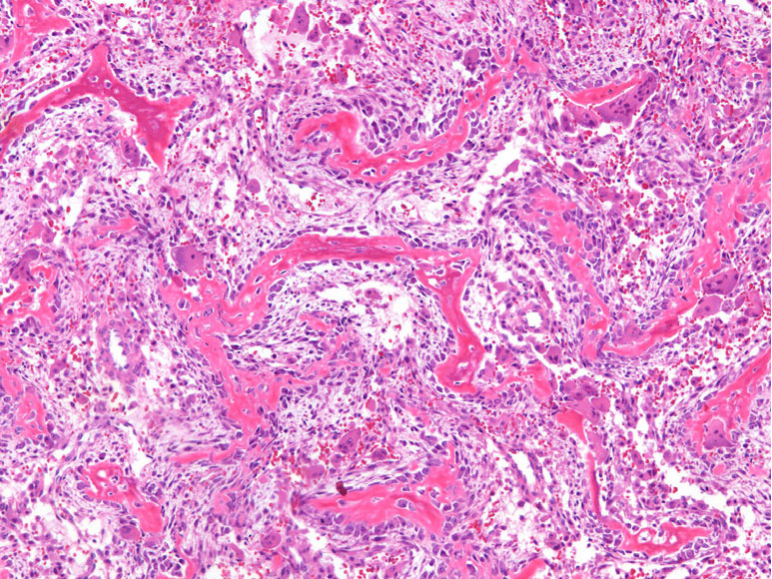
**Permanent section of the left cuboid resembling osteosarcoma**. Area of tumor diagnostic of osteosarcoma in which the tumor cells are large, hyperchromatic, mitotically active, and associated with coarse lace-like woven bone.

Evaluation of the entire tissue specimen revealed a neoplasm that varied in morphology. The majority of the tumor was solid and in regions consisted of randomly interconnecting trabeculae of woven bone rimmed prominently by neoplastic osteoblasts. The plump osteoblasts had abundant eosinophilic cytoplasm and round or oval nuclei with fine chromatin. The intertrabecular spaces were filled with loose vascular connective tissue containing congested capillaries, extravasated red blood cells, and scattered osteoclast-type giant cells. The morphologic features were reminiscent of osteoblastoma (figure 5). In other areas, however, the neoplastic osteoblasts grew in solid aggregates, were cytologically malignant with nuclear enlargement, coarse chromatin, and hyperchromasia, demonstrated many mitoses and produced coarse lace-like neoplastic bone typical of a high grade osteosarcoma (figure 6). Based on the histologic features the tumor was diagnosed as a high grade osteoblastoma-like variant of osteosarcoma.

A staging computed tomography (CT) scan of the chest showed no evidence of metastatic disease. The patient was started on an appropriate chemotherapy regimen and subsequently underwent a below the knee amputation (BKA). Dissection of the cuboid revealed residual small foci of osteosarcoma in the bone neighbouring the cement plug, as well as in the soft tissues immediately adjacent to the bone. No vascular invasion was identified and all margins of resection were widely negative.

## Discussion

Osteosarcoma is a malignant neoplasm with a spectrum of biological potential although most are high grade and behave in an aggressive fashion. This form of cancer usually affects patients in the 2^nd ^half of the 2^nd ^decade of life [1]. When osteosarcoma develops in an older patient, a pre-disposing condition such as Paget's disease of bone or prior radiation exposure to the affected bone should be suspected [4]. Osteosarcoma most commonly arises within the medullary cavity of the metaphyseal region of long bones [5]. The distal femur, proximal tibia, and proximal humerus are most commonly affected.

It is exceedingly uncommon for osteosarcoma to originate in the bones of the foot. In a review of all cases of osteosarcoma from 1911-1992 at the Rizzoli Orthopedic Institute in Italy, the incidence of osteosarcoma in the foot was 0.6%, with 12 identified cases of the foot out of a total of 1,929 osteosarcomas [3]. The calcaneus was most commonly involved, followed by the talus. The most common presenting symptom of osteosarcoma of the foot is pain that is persistent and may be worse at night [1]. Often, the patient may recall a singular event that precipitated the symptoms. Physical exam frequently reveals swelling and pain associated with a mass. Initial evaluation should include an x-ray, followed by a CT scan and/or MRI.

Classically, osteosarcoma appears as a mixed lytic and blastic mass with poorly defined margins, cortical destruction, and a soft tissue mass [1,6]. However, in some cases it may be very difficult to differentiate osteosarcoma from benign lesions such as chondroblastoma, enchondroma, giant cell tumor, osteoblastoma or aneursymal bone cyst, even with the advent of CT and MRI [6,7]. Accordingly, a tissue biopsy is required to establish the diagnosis.

Osteosarcoma of the foot has some clinical features that differ from those associated with osteosarcoma of long bone. In terms of age of presentation, patients with osteosarcoma of the foot are older, with a mean age of diagnosis at 32 years old [2]. In contrast, conventional osteosarcoma is most common in children and adolescents, with the peak age between 15 and 20 years old [3]. In addition, patients with osteosarcoma of the foot commonly have a long delay from the time of onset of symptoms to diagnosis, with the mean interval greater than 2 years [3]. Osteosarcoma of the long bones, however, is usually diagnosed within months of the symptom onset [2]. Lastly, osteosarcomas of the foot generally are lower grade than the typical osteosarcoma affecting long bones [3]. In a study by Biscaglia et al, 42% of the cases involving the foot were low grade, compared to the typical 5-10% of conventional osteosarcomas classified as low grade [3]. In sum, osteosarcomas of the foot differ from osteosarcomas of the long bones in terms of age of presentation, time from symptom onset to diagnosis, and histological grade - all important considerations when evaluating a patient with a suspicious foot lesion.

Osteosarcoma is classified histologically into a variety of different subtypes with the most common being chondroblastic [8]. Fox et al. reported a case of chondroblastic osteosarcoma involving the cuboid bone, which also featured a prominent giant cell component that complicated the initial diagnosis [5]. The osteoblastoma-like variant is a rare form and notable because of its morphologic similarity to osteoblastoma, which is a benign primary bone-forming tumor [9]. The tumor in this case report was considered malignant because of the severe cytologic atypia and high proliferative activity manifested by the tumor cells.

Important distinctions exist between osteoblastoma-like osteosarcoma and conventional osteosarcoma. Bertoni et al. reviewed a series of 11 patients with osteoblastoma-like osteosarcoma, and found that the average age of presentation was older at 29 years of age [10]. The lesions were low-grade, but had a high rate of recurrence if adequate surgical margins were not achieved. The most common location was the tibia followed by the spine. Of note, the authors also found that osteoblastoma-like osteosarcoma involved bones not commonly affected by conventional osteosarcoma, including the small bones of the skull and face.

This case report, in summary, presents the rare diagnosis of osteoblastoma-like variant of osteosarcoma arising in the cuboid bone which, to the best of our knowledge, is the first published case. This report highlights the importance of tissue biopsy in the evaluation of suspicious foot lesions, the limitations of imaging studies, and the challenges of pathologic analysis in establishing a diagnosis when elements of both benign and malignant etiologies co-exist.

## Competing interests

The authors declare that they have no competing interests.

## Authors' contributions

NLK conceived the idea and wrote the paper.

AER was responsible for editing and approving the final manuscript

KAR was responsible for editing and approving the final manuscript.

All authors read and approved the final manuscript.

## Consent

Written informed consent was obtained from the patient for publication of this case report and any accompanying images. A copy of the written consent is available for review by the Editor-in-Chief of this journal.
